# Differential Tear Lipid Metabolomics Signatures Discriminate Superior Limbic Keratoconjunctivitis From Dry Eye Disease

**DOI:** 10.1167/tvst.14.11.37

**Published:** 2025-11-26

**Authors:** Yan Zong, Sihao Liu, Yubin Yu, Chunxiao Wang, Chao Cheng, Xiuping Liu, Jiangbo Du, Yonghui Gu, Kaili Wu

**Affiliations:** 1 Zhongshan Ophthalmic Center, State Key Laboratory of Ophthalmology, Sun Yat-Sen University, Guangdong Provincial Clinical Research Center for Ocular Diseases, Guangzhou, People's Republic of China; 2 Department of Ophthalmology, Suzhou Municipal Hospital, The Affiliated Suzhou Hospital of Nanjing Medical University, Suzhou, Jiangsu, People's Republic of China; 3 Department of Epidemiology, Center for Global Health, School of Public Health, Nanjing Medical University, Nanjing, Jiangsu, People's Republic of China

**Keywords:** superior limbic keratoconjunctivitis (SLK), dry eye disease (DED), metabolomics, tear fluids, secretory phospholipase A2 (sPLA2)

## Abstract

**Purpose:**

Superior limbic keratoconjunctivitis (SLK) and dry eye disease (DED) exhibit similar clinical manifestations, complicating diagnosis. We aimed to compare tear metabolomic profiles and secretory phospholipase A2 (sPLA2) levels in patients with SLK versus patients with DED.

**Methods:**

We established a cross-sectional study involving 56 subjects: 20 patients with SLK, 21 patients with DED, and 15 matched healthy controls (solely for measuring sPLA2). Tear fluids collected via Schirmer strips underwent liquid chromatography with tandem mass spectrometry metabolomic analysis. Metabolites were quantitatively analyzed and matched to metabolic pathways and biomarkers. Metabolic differences between patients with SLK and patients with DED were identified through multivariate statistical analysis. Western blot measured the content of sPLA2 in tears of the three groups.

**Results:**

Tear analysis annotated 274 metabolites. Twenty-three metabolites showed significant differences (*P* < 0.05) between the 2 groups, comprising 20 increased and 3 decreased in SLK. Top differential metabolic pathways were glycerophospholipid, alpha-linolenic acid, and linoleic acid metabolism. Receiver operating characteristic (ROC) analysis showed that five metabolites could be considered as potential biomarkers for distinguishing SLK from DED. Tears PLA2 was significantly higher in SLK than healthy controls (*p* < 0.05), but without difference between SLK and DED.

**Conclusions:**

The study revealed distinct metabolomic profiles in SLK versus DED tears, with notable changes in glycerophospholipid metabolism and elevated sPLA2 levels in SLK.

**Translational Relevance:**

Tear metabolomics distinguishes SLK from DED at the molecular level through dysregulated glycerophospholipid metabolism. In SLK, compared with controls, this dysregulation coincides with elevated sPLA2, an enzyme hydrolyzing glycerophospholipids, indicating lipid-driven inflammation.

## Introduction

Superior limbic keratoconjunctivitis (SLK) is a chronic eye condition characterized by inflammation of one or both eyes that can persist for years. It was first described in the 1950s and officially named in 1963.^1^^,^^2^ SLK predominantly affects individuals aged 20 to 70 years old, with the most common age range being 40 to 50 years old.^3^^,^^4^ Clinical symptoms of SLK include foreign body sensation, burning, dryness, and photophobia. The exact cause of SLK is not fully understood, but it is thought to be related to mechanical injury,^5^^,^^6^ tear film instability,^7^ and autoimmune factors.^8^^,^^9^ Due to the lack of standardized diagnostic criteria and effective treatment methods, diagnosing and treating SLK is challenging.

The symptoms and signs of SLK are similar to those of dry eye disease (DED), often leading to SLK being overlooked or misdiagnosed as DED. Whereas there is a correlation between the pathogenesis of the two conditions, there are also distinct differences. Studies have shown that patients with DED have an increased blink rate,^7^ and blink-related microtrauma is one of the pathogenic mechanisms of SLK.^10^ Certain matrix metalloproteinases (MMPs), such as MMP-9,^11^ are used to diagnose inflammation related to DED. However, MMP-1 and MMP-3 are overexpressed in SLK, whereas MMP-9 is not detected in SLK.^12^ Clinically, medications used to treat DED have also been found effective for SLK.^13^ In summary, there are both correlations and differences between the two conditions, and further research is needed to explore their relationship.

Our previous study has applied untargeted metabolomics to the SLK group to demonstrate the unique metabotypes and identified significant alterations in tear metabolites compared the to healthy control group. The results revealed a possible change of lipid metabolism in patients with SLK, especially the ω-3 and ω-6 polyunsaturated fatty acids (PUFAs) balance, which indicated the potential inflammatory process in the pathogenesis of the disease.^14^ Researchers studying the metabolic profile of patients with DED have discovered that decreased levels of glycerophosphocholine are associated with the condition.^15^^,^^16^ Additionally, there is a significant reduction in the concentrations of carnitine, L-acetylcarnitine, and L-propionylcarnitine in the tear fluids of patients with DED.^17^ Moreover, after supplementation with essential PUFA, the levels of choline and acetylcholine in tears were found to have increased in patients with DED.^18^ Given the symptomatic and clinical similarities between SLK and DED, further research and additional indicators are necessary to differentiate the tear fluids in SLK and DED.

Lipid-related protein secretory phospholipase A2 (sPLA2), a lipid hydrolase enzyme, plays a key role in lipid metabolism and the regulation of pro-inflammatory factors. SPLA2 is associated with important physiological and biochemical processes within the body. It can exhibit multiple cellular functions,^19^ including acting as an innate ocular surface barrier against microbial infection^20^^,^^21^ and playing a role in regulating inflammatory responses. The sPLA2-IIa activity in the tears of patients with DED has been reported to be significantly higher than in tears of healthy subjects.^22^ Our research group has observed an increase in the content of sPLA2-IIa in the tears of patients with allergic conjunctivitis.^23^

This study aims to analyze tear fluid metabolomics in patients with SLK and patients with DED, identify unique metabolites, and investigate biological pathways to find diagnostic biomarkers. We also assessed the expression and content changes of sPLA2 to support our previous findings and advance the understanding of SLK’s pathogenesis, diagnosis, and treatment.

## Methods

### Study Subjects

From May 2021 to September 2022, a total of 20 patients with SLK and 21 patients with DED were recruited at Zhongshan Ophthalmic Center, a tertiary eye hospital in Guangzhou, China. Another 15 healthy controls were recruited specifically for sPLA2 measurement to provide a definitive biochemical baseline for this key inflammatory enzyme, thereby validating the disease phenotype of our SLK cohort. Our limited tear volume resources were then prioritized for the novel metabolomic comparison between SLK and DED, the primary focus of this study. All subjects were selected to obtain age- and sex-matched study cohorts. This study was conducted in accordance with the Declaration of Helsinki (World Medical Association, 2013) and approved by the Institutional Review Board of the Zhongshan Ophthalmic Center (No: 2018KYPJ140).

Patients with SLK were diagnosed based on their medical history, symptoms, and signs after routine eye examinations. For the consistency of our series study, the inclusion criteria and exclusion criteria were followed with our previous research.^14^ Based on the summary of SLK’s clinical features,^3^ we included patients with moderate to severe SLK as study participants, with superior limbus/conjunctival staining (SCS) as the main judging index. Patients with an SCS score of 2 or 3 were ultimately enrolled in the study.

The patients with DED were diagnosed based on their medical history, symptoms, and signs after routine eye examinations. The inclusion criteria were set according to the consensus of diagnosis and treatment of DED by the Cornea Group of the Chinese Ophthalmological Society.^24^ Patients with mild and moderate DED were recruited. To collect enough tear volume, the Schirmer’s test (SIT) should be ≥2 mm. Exclusion criteria included any of the following conditions: (1) history of eye surgery; (2) other eye conditions that can cause DED symptoms, such as blepharitis, meibomian gland dysfunction, pterygium and allergic conjunctivitis; (3) a history of eye topical therapy or use of contact lenses within the previous 3 months; (4) inflammatory and infectious eye diseases within the past 6 months; and (5) inflammatory or autoimmune systemic diseases such as rheumatism, Sjogren’s syndrome, or other diseases affecting tear secretion.

Healthy controls were randomly recruited based on the following criteria: (1) no history of chronic eye diseases in the past 6 months; (2) no local eye treatments or contact lens use in the past 3 months; and (3) absence of systemic diseases, such as rheumatism, Sjögren’s syndrome, or other conditions affecting tear secretion.

For all participants, the basic demographic data, including age, gender, and medical history, was recorded and ocular examinations, including visual acuity, intraocular pressure, slit-lamp and fundus examination, tear breakup time (TBUT), corneal fluorescein staining, and SIT were performed.^23^^,^^25^ One eye of each subject was randomly selected for statistical analysis.

### Sample Collection

Tear collection followed our previous research methods.^14^ The moistened area length was measured on the SIT strip to quantify the tear fluid volume. Samples with less than 2 mm or reflex tears were excluded. Each filter strip was placed in a 1.5 mL microtube (Axygen, Jiangsu, China) and stored at −80°C. For patients with SLK or DED with consistent severity in both eyes, tear samples from both eyes were combined if the tear volume was low.

### Metabolites Extraction and Liquid Chromatography Tandem Mass Spectrometry Analysis

Metabolites were extracted from tear-soaked Schirmer strips. Briefly, the strips were cut into 2 to 3 mm pieces and placed in 0.22 µm Ultrafree-MC Filter Devices (Merck Millipore, Boston, MA, USA). Each device was prefilled with ultra-pure water (20 times the volume of tears absorbed by the strips, calculated based on 7 µL of tears per 10 mm of wetted test paper). After incubation for 30 minutes on ice, the devices were centrifuged at 13,800×*g* for 15 minutes at 4°C to collect the filtrate.

An aliquot of 100 µL of the filtrate was transferred to a new tube and mixed with 400 µL of extraction solution (acetonitrile:methanol = 1:1, v/v, containing a mixture of isotopically labeled internal standards). The mixture was vortexed for 30 seconds, sonicated for 10 minutes in an ice-water bath, and then incubated at –40°C for 1 hour to precipitate proteins. Subsequently, the sample was centrifuged at 13,800×*g* for 15 minutes at 4°C. The resulting supernatant was transferred to a fresh glass vial for liquid chromatography tandem mass spectrometry (LC-MS/MS) analysis. A quality control (QC) sample was prepared by combining equal aliquots of the supernatant from all individual samples.

LC-MS/MS analyses were performed using a Vanquish UHPLC system (Thermo Fisher Scientific) coupled to an Orbitrap Exploris 120 mass spectrometer (MS; Thermo Fisher Scientific). Chromatographic separation was achieved on a Waters ACQUITY UPLC BEH Amide column (2.1 mm × 100 mm, 1.7 µm) maintained at 4°C. The mobile phase consisted of (A) 25 mmol/L ammonium acetate and 25 mmol/L ammonium hydroxide in water (pH = 9.75) and (B) acetonitrile. The injection volume was 2 µL.

The MS was operated in information-dependent acquisition (IDA) mode under the control of Xcalibur software (Thermo Fisher Scientific). The electrospray ionization (ESI) source conditions were set as follows: sheath gas flow rate, 50 arb; auxiliary gas flow rate, 15 arb; capillary temperature, 320°C; spray voltage, 3.8 kV (positive mode), or –3.4 kV (negative mode). Full MS scans were acquired at a resolution of 60,000, and tandem MS/MS scans were acquired at a resolution of 15,000. A stepped normalized collision energy (NCE) of 10, 30, and 60 eV was applied.

### Metabolites Identification and Data Processing

The raw data were converted to mzXML format using ProteoWizard and processed with a custom R software package via XCMS. Metabolites were matched against databases like the Human Metabolome Database (HMDB).

Following our previous methods, all metabolite data were normalized by log2 transformation. Principal component analysis (PCA), orthogonal partial least square-discriminant analysis (OPLS-DA), and volcano plot analysis were performed using Simca-P version .14.1 software (Umetrics AB) to reveal data structure and identify differential metabolites. Variable importance in the projection (VIP) was calculated using the OPLS-DA model. Criteria for selecting differential metabolites were selected based on MS2 score > 0.5, VIP > 1, and P (*t*-test) < 0.05. Bar charts were generated using GraphPad Prism 8 (GraphPad Software, Inc., 2018, La Jolla, CA, USA). Metabolic pathway topology analysis and receiver operating characteristic curves (ROC) were performed using MetaboAnalyst version 5.0 to identify relevant metabolic pathways and evaluate a potential combined biomarker model.

### Protein Quantifying and Western Blot Analysis

We detected the expression of sPLA2 in tears through Western blot analysis, which was performed on tear samples from patients with SLK, DED, and healthy controls, adhering to the reported protocol.^26^ Due to the limited volume of tear samples, we utilized SDS-PAGE and Coomassie Blue staining to estimate the protein content in the samples. Band density quantification allowed us to compare the expression of sPLA2 across different samples.^27^ After excluding samples that were depleted in the metabolomics analysis or had volumes exceeding the gel wells’ capacity, we allocated the remaining 15 SLK samples and 15 DED samples. These were matched with 15 healthy volunteers, forming 3 groups. Concurrently, the wetting length of the Schirmer’s test strips was approximately equivalent in each group.

The procedure involved washing Schirmer strips as previously described to obtain samples. These samples were then prepared with SDS–PAGE sample buffer, heated, and loaded onto two parallel gels. After electrophoresis, proteins from one gel were transferred to PVDF membrane. The membrane was blocked and then incubated with the following antibodies: anti-sPLA2 (14 kDa) rabbit monoclonal primary antibody (1:1,000; Catalog #52568, Signalway Antibody LLC/SAB, College Park, MD) and an appropriate horseradish peroxidase (HRP)-conjugated secondary antibody. Protein bands were visualized using chemiluminescence. Meanwhile, the other gel was stained with Coomassie Blue. Band density was quantified using ImageJ software (National Institutes of Health, Bethesda, MD), with target protein values against total proteins on the stained gel.

### Statistical Analysis

All statistical analyses were performed using SPSS software (version 26.0; IBM, Armonk, NY). Normality test was performed for numerical data first. The results for numerical variables were presented as the mean ± standard deviation or median (interquartile range), and results for categorical variables were presented as the number (percent). The comparison of means of numerical variables between the two groups were evaluated using the Student’s *t*-test or the Mann-Whitney *U* test. Comparison of means among multiple groups for numerical variables was evaluated by analysis of variance or Kruskal-Wallis test. Chi-square test was used for comparison of categorical variables among groups. *P* values less than 0.05 were considered statistically significant.

## Results

### Demographics and Ocular Parameters

A total of 56 subjects participated in this study. The SLK group consisted of 20 patients (mean age = 42.90 ± 13.42 years, 60.00% female patients), the DED group included 21 patients (mean age = 39.76 ± 12.71 years, 61.90% female patients), and the healthy control group, used solely for measuring sPLA2, comprised 15 volunteers (mean age = 38.10 ± 10.44 years, 53.33% female patients; Table 1).There were no statistically significant differences in sex proportions, ages, or SIT scores among the three groups (*P* = 0.87, *P* = 0.5, and *P* = 0.08, respectively). However, there were significant differences in TBUT and tear volume in Schirmer strips among the three groups (*P* = 0.00 and *P* = 0.01, respectively).

**Table 1.** Demographic Characteristics of Subjects in Different Groups

Feature	DED (*n* = 21)	SLK (*n* = 20)	Health (*n* = 15)	Overall *P* Value	Multiple Mean Comparisons
Gender, female, %	13, 61.90	12, 60.00	8, 53.33	0.87	–
Age, y	39.76 ± 12.71	42.90 ± 13.42	38.10 ± 10.44	0.50	–
TBUT, s	3.33 ± 0.66	3.28 ± 1.21	5.93 ± 1.28	0.00	Health > DED; Health > SLK
SⅠT (IQR), mm	7.00 (4.25, 9.75)	5.25 (2.75, 7.13)	8.00 (3, 13)	0.08	–
Tear volume (IQR), µL	8.95 (6.28, 11.63)	5.05 (3.78, 6.33)	6.51 (2.62, 10.4)	0.01	Health >SLK; DED > SLK

DED, dry eye disease; IQR, interquartile range; SⅠ,: Schirmer I test; SLK, superior limbic keratoconjunctivitis; TBUT, tear break-up time.

### Overall Metabolites of Tear Samples

Through data processing and MS peak identification, a total of 274 metabolites were annotated with a cutoff value of 0.3 in both groups of samples, including 191 metabolites derived from positive ionization modes and 83 metabolites derived from negative ionization modes. Among them, 102 metabolites with an MS2 score > 0.8 were identified, including 93 metabolites in positive ion mode and 9 metabolites in negative ion mode. Additionally, 225 metabolites with an MS2 score > 0.5 were identified, including 152 metabolites in positive ion mode and 73 metabolites in negative ion mode. These 225 metabolites were selected for further analysis. Most of the identified metabolites were lipids, followed by amino acids and nucleotides.

### Metabolomics Analysis of Differences Between SLK and DED Groups

**Figure 1.**
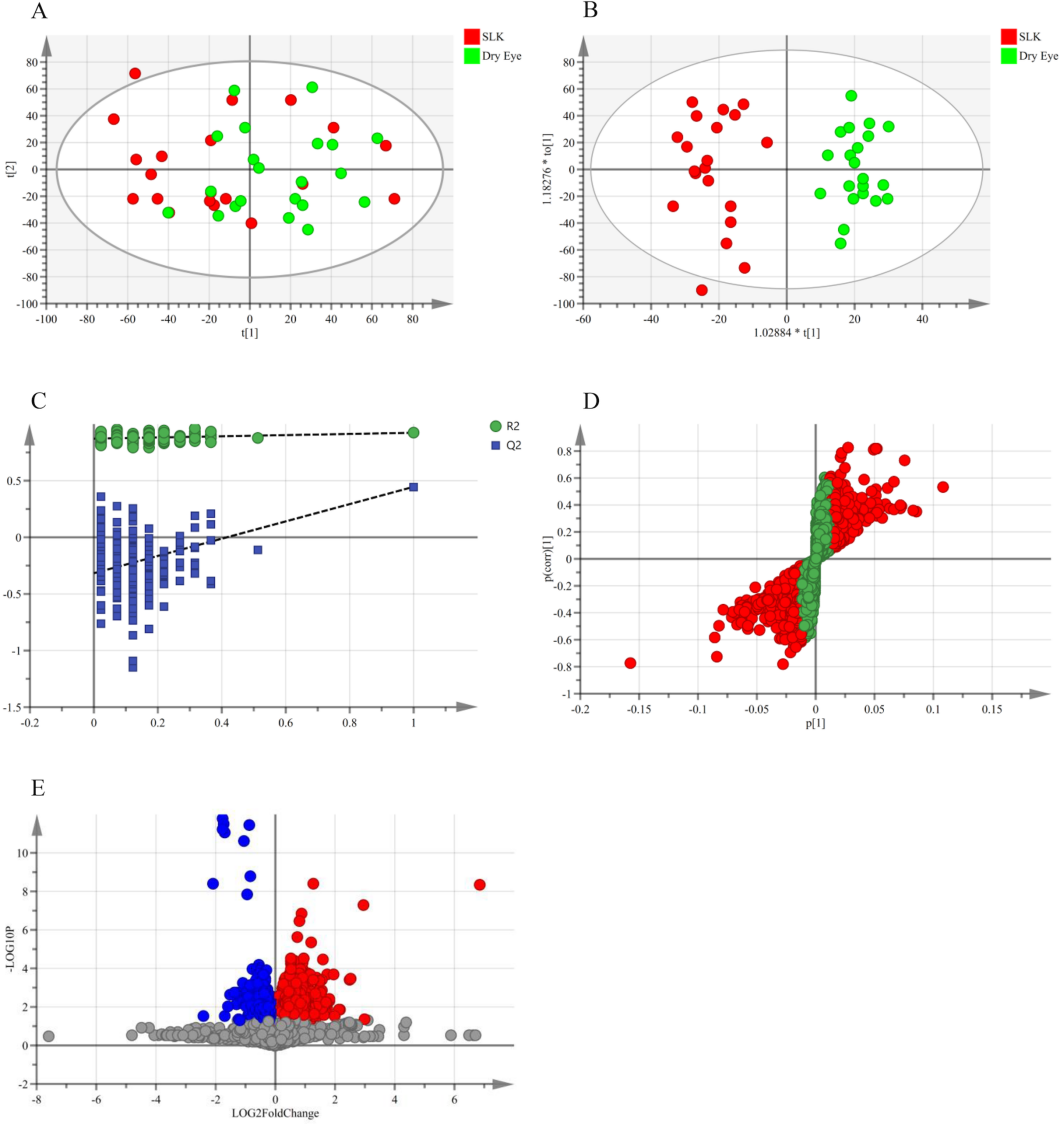
**Metabolomic analysis of tear samples from patients with SLK and DED**. (**A**) Principal component analysis (PCA) score plots for the SLK (*red*) and DED (*green*) groups. The X-axis (t[1]) and Y-axis (t[2]) indicate the first and second principal components, respectively. Each point represents a sample. (**B**) Orthogonal partial least square-discriminant analysis (OPLS-DA) score plots for the SLK (*red*) and DED (*green*) groups. The x-axis (t[1]) represents the predicted principal component score of the first principal component, showing the inter-group differences of the samples. The y-axis (to[1]) represents the orthogonal principal component score, showing the intra-group differences of the samples. (**C**) Permutation test plot: the closer the original model R2Y is to 1, the more the established model conforms to the true situation of the sample data; at the same time, as the permutation retention degree gradually decreases, the proportion of the permuted Y variable increases, and the Q2 of the random model gradually decreases. This indicates that the original model has good robustness and does not have overfitting. (**D**) S-plot: the *red dots* indicate that the VIP values of these metabolites are ≥1, and the *green dots* indicate that the VIP values of these metabolites are < 1. (**E**) Volcano plot showing metabolites that were significantly increased (*red*) or decreased (*blue*) in tears from patients with SLK compared to the patients with DED group.

First, an unsupervised PCA was performed to obtain an overview of the metabolic profiles. The PCA score plot (Fig. 1A) showed a trend of separation between the SLK and DED groups; however, there was considerable overlap between them. This indicates that the largest sources of variation within the dataset may not be primarily driven by the diagnostic classification and that unsupervised analysis could not clearly distinguish between the two conditions. To identify the robust metabolic differences between SLK and DED that are consistent across patients, we used a supervised OPLS-DA model. This method efficiently separates the variation in the data into two parts: (1) class-related variation (predictive component) and (2) class-unrelated variation (orthogonal components), thereby isolating biologically relevant group-specific signals. The OPLS-DA model revealed clear separation between the SLK and DED groups (Fig. 1B), in stark contrast to the PCA result. To rigorously validate this model and rule out overfitting, a permutation test (200 iterations) was performed. The results (Fig. 1C) demonstrated excellent robustness: high explained variance (R2Y = 0.923) and high predictive ability (Q2 = 0.446). Crucially, the negative Q2 intercept (–0.317 < 0) and the positive slopes of the regression lines for both R2 and Q2 confirm that the original model is valid and not a result of overfitting. Figure 1D shows the S-plot of OPLS-DA, used for variable selection based on VIP values. Based on the criteria of VIP > 1 and *P* < 0.05 (*t*-test), the results of differential metabolites selection were visualized in the form of a volcano plot, as shown in Figure 1E.

The VIP score was used to aid in the selection of significantly differential metabolites, as higher VIP scores indicate greater importance of the metabolite for classification. Combining VIP scores with statistical test *P* values, we identified 23 significantly changed metabolites, which were helpful in distinguishing between the SLK and DED groups. Based on fold change, 20 metabolites were found to be significantly increased in patients with SLK compared with patients with DED, whereas 3 metabolites were significantly decreased (Table 2).

**Table 2.** Basic Information of 23 Differential Metabolites Between the SLK Group and the DED Group

MS2.name	Trend	FC (SLK/DED)	VIP	*P* Value	SLK Mean ± SD	DED Mean ± SD
PE(22:2/14:1)	↑	2.44	2.15	4.13 E-03	(11.06 ± 9.15) E-04	(4.52 ± 3.53) E-04
Humulinic acid A	↑	1.86	1.36	6.15 E-03	0.005 ± 0.003	0.003 ± 0.001
3-Hydroxyisovalerylcarnitine	↑	1.86	1.82	1.59 E-02	0.009 ± 0.007	0.005 ± 0.004
Terephthalic acid	↑	1.80	1.30	6.59 E-03	0.280 ± 0.185	0.155 ± 0.073
6-(1-Hydroxyethyl)-2,2-dimethyl-2H-1-benzopyran	↑	1.75	2.13	3.64 E-03	0.051 ± 0.028	0.029 ± 0.015
Alpha-dihydroartemisinin	↑	1.72	1.38	4.10 E-03	0.013 ± 0.007	0.007 ± 0.003
Sterigmatocystin	↑	1.68	1.17	1.39 E-02	0.002 ± 0.001	0.001 ± 0.001
L-Acetylcarnitine	↑	1.64	1.28	2.09 E-03	0.533 ± 0.265	0.324 ± 0.116
PC(22:2/14:1)	↑	1.63	1.24	1.69 E-02	0.021 ± 0.013	0.013 ± 0.008
Sodium 3-(3,4-dihydroxyphenyl)-2-hydroxypropanoate	↑	1.63	1.22	4.31 E-03	0.115 ± 0.060	0.071 ± 0.030
2,4-Dihydroxybenzoic acid	↑	1.60	1.31	2.04 E-03	0.104 ± 0.046	0.065 ± 0.027
PC(18:1/14:0)	↑	1.59	1.11	3.18 E-02	(16.76 ± 11.34) E-04	(10.54 ± 5.81) E-04
AF Toxin II	↑	1.56	1.05	3.69 E-03	0.025 ± 0.012	0.016 ± 0.006
LysoPC(16:1)	↑	1.55	1.88	4.97 E-02	(9.22 ± 6.97) E-04	(5.93 ± 2.52) E-04
Succinic anhydride	↑	1.54	1.28	2.25 E-02	0.016 ± 0.010	0.010 ± 0.004
Creatine	↑	1.52	1.21	1.27 E-02	0.160 ± 0.078	0.106 ± 0.053
1,3-Benzenediol	↑	1.50	1.01	2.13 E-02	0.451 ± 0.241	0.300 ± 0.152
3-Phenoxybenzoic acid	↑	1.49	1.01	2.26 E-02	0.186 ± 0.099	0.124 ± 0.063
5-Aminopentanal	↑	1.49	1.18	3.82 E-05	0.014 ± 0.004	0.009 ± 0.002
Choline	↑	1.30	1.01	4.87 E-02	0.074 ± 0.030	0.057 ± 0.024
Testosterone	↓	0.80	1.14	2.95 E-02	0.005 ± 0.002	0.006 ± 0.002
Hydroxyprolyl-Leucine	↓	0.69	1.50	6.18 E-03	(9.11 ± 4.51) E-04	(13.26 ± 4.66) E-04
2-Propionylpyrrole	↓	0.68	1.21	2.90 E-03	0.030 ± 0.015	0.045 ± 0.014

FC, fold change (SLK/DED); m/z, mass-to-charge ratio; rt, retention time; VIP, variable importance in the projection.

Metabolites were identified by MS/MS matching. Differential metabolites were filtered by VIP > 1 and *P* < 0.05 (*t*-test). Direction of change (↑ increase and ↓ decrease) is relative to the DED group.

### Metabolite Set Enrichment and Pathway Analysis

**Figure 2.**
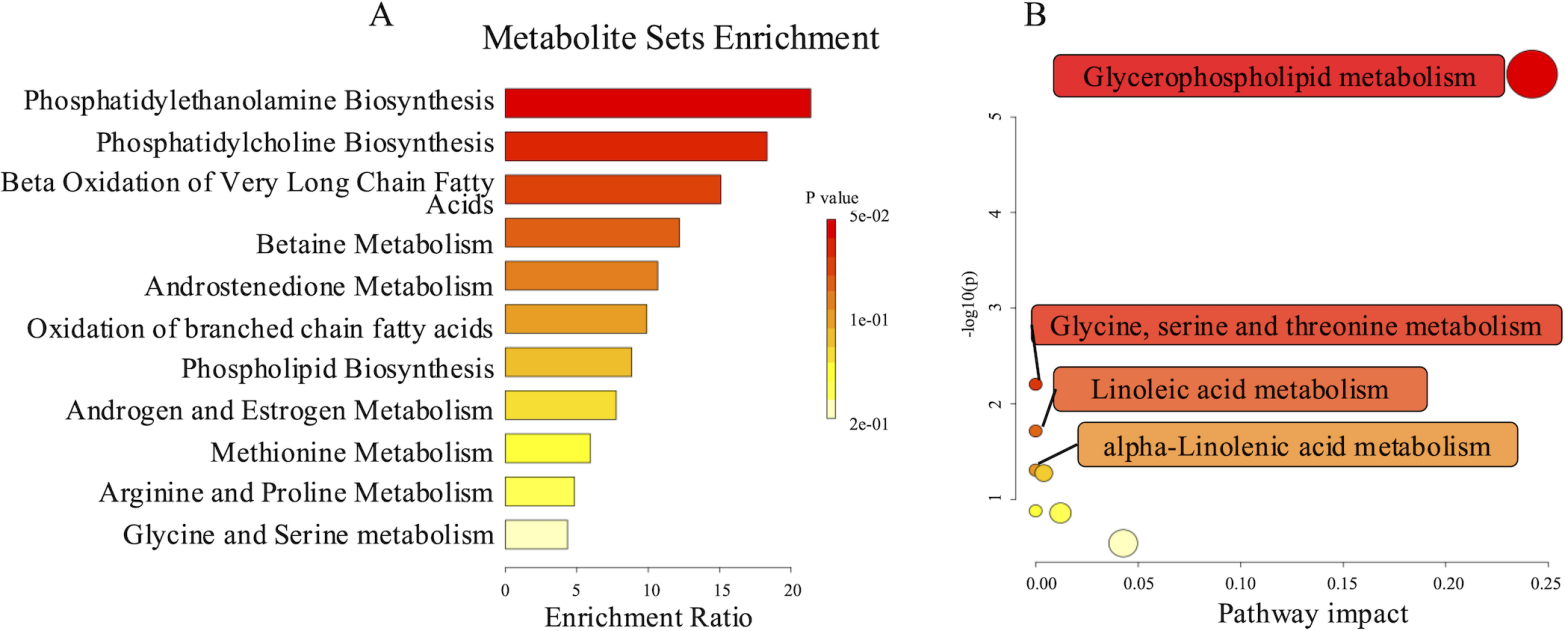
**Metabolite set enrichment and pathway analysis of changed metabolites in tear fluids of patients with SLK compared to DED.** MetaboAnalyst (http://www.metaboanalyst.ca) was used to perform metabolite set enrichment analysis (**A**) and pathway analysis (**B**) for 23 significantly changed metabolites. According to the significance of each pathway, varying colors were used to mark the main involved metabolic processes.

To interpret the differentially expressed metabolites and further investigate the associated metabolic pathways, the 23 significantly changed metabolites, which were potentially involved in the SLK pathological mechanisms, were subjected to set enrichment and pathway analysis. Figure 2 shows the metabolite enrichment and metabolic pathway analysis which are biologically significant in selected databases (Small Molecule Pathway Database [SMPDB] and Kyoto Encyclopedia of Genes and Genomes [KEGG]). These altered metabolites primarily referred to glycerophospholipid metabolism, glycine, serine, and threonine metabolism, alpha-linolenic acid (ALA) metabolism, linoleic acid (LA) metabolism, and beta-oxidation of very long chain fatty acids. Among them, glycerophospholipid metabolism pathway was the most significant.

### Screening of Potential Biomarkers

**Figure 3.**
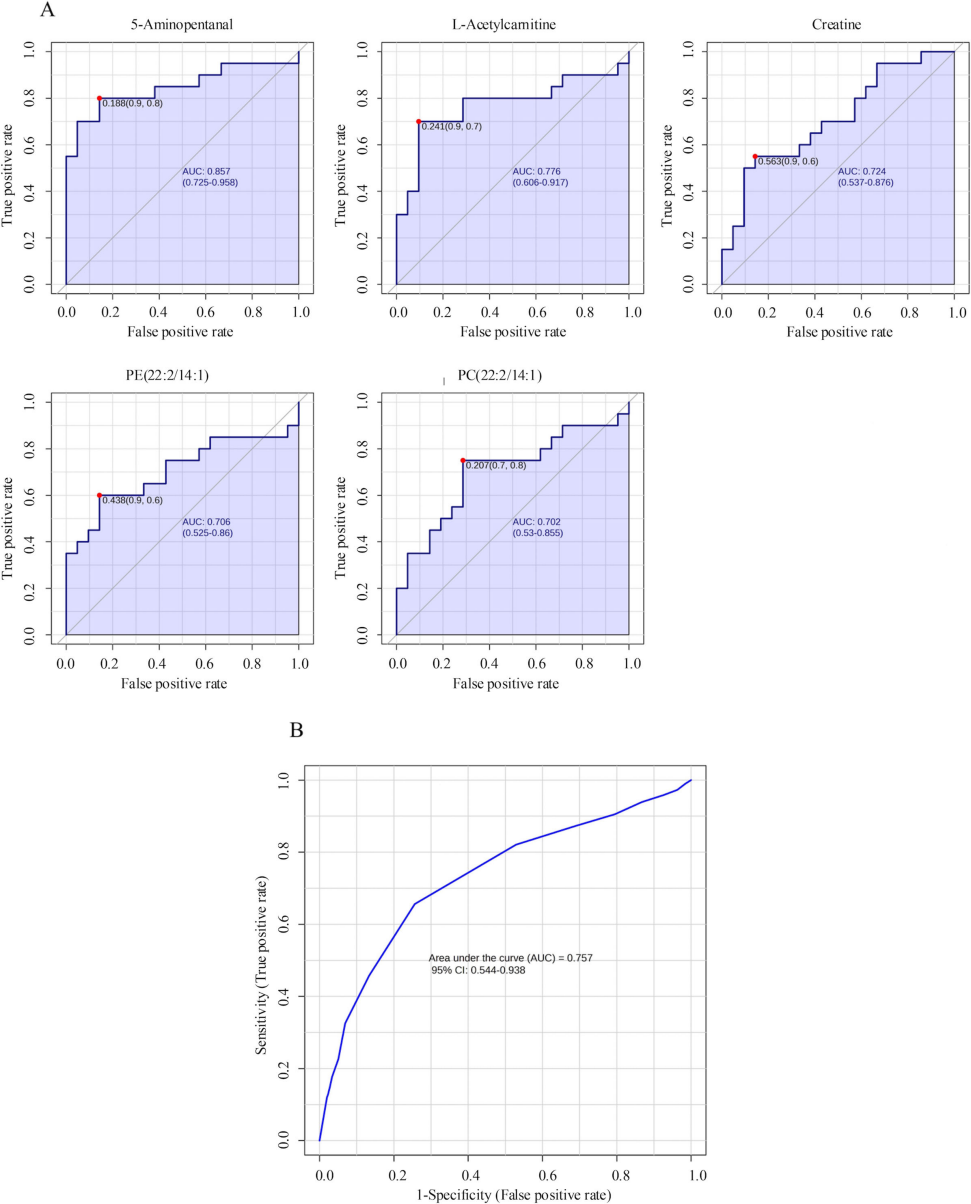
**Receiver operating characteristic (ROC) curve analysis was performed on** **five** **metabolites that changed in SLK tear fluids, in comparison to those in DED.** (**A**) Using ROC curves of altered tear metabolites to evaluate diagnostic efficacy of distinguishing SLK individuals from DED individuals. The AUCs of 5 metabolites were all ≥0.7, indicating good predictive ability. (**B**) The evaluation model of combining five metabolites can effectively distinguish the SLK group from the DED group (AUC = 0.76). Note: PE(22:2/14:1) is a phosphatidylethanolamine, and PC(22:2/14:1) is a phosphatidylcholine.

Receiver operating characteristic (ROC) curve analysis is generally considered to be the gold standard for the assessment of biomarker performance. Based on VIP, *P* value, fold change, peak intensity, literature reviews, and biological significance, five potential biomarkers with high sensitivity (true positive rate) and specificity (true negative rate) were selected. The areas under the ROC curves (AUCs) for the metabolites identified in this study are as follows: 5-aminopentanal (0.86), L-acetylcarnitine (0.78), creatine (0.72), PE(22:2/14:1; a phosphatidylethanolamine [PE]; 0.71), and PC(22:2/14:1; a phosphatidylcholine [PC]; 0.70), as shown in Figure 3. Furthermore, an ROC curve-based biomarker model was established to evaluate the predictive power of the combination of the five metabolites (see Fig. 3B). These data showed that the highest confidence in differentiating SLK subjects from DED subjects was achieved with an AUC value of 0.76. Therefore, one or more of these five metabolites can be considered as potential biomarkers associated with SLK that differentiate it from DED.

### sPLA2 Changed in Tears of Patients With SLK 

**Figure 4.**
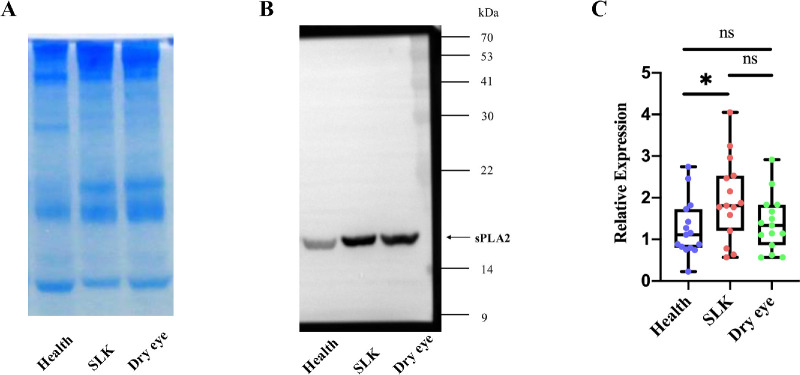
**Quantification of sPLA2 in tears of** **patients with** **SLK** **(*n* = 15),** **patients with dry eye** **(*n* = 15),** **and healthy controls (*n* = 15).** (**A**) SDS–PAGE with Coomassie Blue staining to display the protein bands of tear samples. (**B**) Western blot analysis of sPLA2. A representative chemiluminescence image (merge of the immunoblot signal and the pre-stained molecular weight markers) is shown. Molecular size markers (in kDa) are indicated on the right. A specific immunoreactive band for sPLA2 is detected at the expected size of approximately 14 kDa, as indicated by the *arrow*. (**C**) Normalized expression means the relative content of sPLA2 was represented by dividing the densitometric value of sPLA2 by the respective total tear protein concentration in gel. Statistically significant differences were marked by an *asterisk* (*P* < 0.05).

Because sPLA2 is a crucial enzyme in the glycerophospholipid metabolism pathway, measuring its concentration in tear fluid can provide additional evidence of metabolic alterations. We conducted a detailed quantitative analysis of sPLA2 levels in the tears of patients with SLK, patients with DED, and 15 healthy controls. The sPLA2 were normalized against tear protein concentration to accurately compare across individuals. In tear fluids, sPLA2 content was higher in patients with SLK than in healthy controls (Fig. 4; *P* < 0.05). Although, the mean level of sPLA2 in SLK tears (1.96 ± 0.98) was higher than that in patients with DED (1.39 ± 0.66), and the mean level of sPLA2 in patients with DED was higher than that in healthy control group (1.25 ± 0.68), but these differences were not statistically significant after 1-way ANOVA (*P* > 0.05).

## Discussion

The controversy between SLK and DED has long persisted due to their similar symptoms and signs, making it difficult to differentiate between them. When these two diseases occur simultaneously, the presence of SLK and other friction-related diseases can aggravate DED symptoms. Additionally, insufficient tear volume and meibomian gland dysfunction (MGD), both causes of DED, are also considered to be associated with SLK.^28^ Our previous data, based on tear and blood studies, revealed a potential lipid metabolism disorder in patients with SLK compared with healthy individuals. Specifically, we observed abnormalities in LA metabolism and the biosynthesis of unsaturated fatty acids, along with alterations in the balance of ω-3 and ω-6 PUFA.^14^^,^^29^ Published reports have indicated that lipid metabolism abnormalities are present in the tears of patients with DED^30^ and that PUFA plays a role in the signs of DED.^31^ To explore the differences and connections between these two conditions, we applied metabolomics to investigate their relationship, focusing on lipid metabolism. The results revealed that the tear metabolome of patients with SLK is distinct from that of patients with DED. By combining VIP, *P*, and MS2 scores, we identified 23 significantly differential metabolites and 4 differential pathways mapped by these metabolites. Among these, three pathways were related to lipid metabolism: LA metabolism, ALA metabolism, and glycerophospholipid metabolism. Combining the current study with our previous two metabolomic analyses of patients with SLK, these findings are mutually corroborative and supportive, all demonstrating significant alterations in lipid-related metabolism.

Notably, the recurrent dysregulation of LA metabolism across our series of studies highlights its potential central role in SLK pathogenesis, distinguishing it from both healthy controls and patients with DED, prompting a deeper mechanistic hypothesis. The key may lie not just in the imbalance of ω-6 and ω-3 PUFAs, but in the specific fate of LA-derived arachidonic acid (AA)^32^ on the ocular surface. We hypothesize that chronic mechanical irritation of the superior bulbar conjunctiva—a hallmark of SLK^28^^,^^33^—triggers a pathological overexpression of sPLA2 (which we found elevated), leading to excessive release of AA from membrane phospholipids.^34^ The subsequent metabolism of this AA pool likely shifts decisively toward pro-inflammatory eicosanoids (e.g., specific hydroxyeicosatetraenoic acids [HETEs] or leukotrienes) via enzymes like 12/15-lipoxygenase,^35^ potentially overwhelming the synthesis of pro-resolving mediators. This self-perpetuating cycle of mechanically induced inflammation, amplified by a specific LA-AA-eicosanoid axis, could directly explain the chronic inflammation, tissue damage, and hyperemia characteristic of SLK. This proposed mechanism moves beyond a general inflammatory state and offers a more precise explanation for why LA metabolism emerges so prominently in SLK compared to DED, where the inflammatory drivers may be more diverse and less tied to this specific pathway. Our findings thus not only expand the understanding of SLK in the PUFA field but also pinpoint a potential mechanistic target for future therapeutic intervention.

Furthermore, the newly discovered differential changes in glycerophospholipid metabolism open another aspect of distinguishing SLK from DED. Glycerophospholipids, which constitute the majority of the tear film’s polar lipid layer,^36^ are essential for maintaining tear film stability.^37^ Various glycerophospholipids are also suggested to play roles in regulating inflammation and oxidative stress, which are common in ocular surface diseases. In this pathway, four important differential metabolites were identified: choline, PC, LysoPC(16:1; a lysophosphatidylcholine [LPC]), and PE. The role of choline in ocular surface disease appears to be complex and condition-specific. Although choline deficiency has been implicated in the pathogenesis of DED due to its crucial role in maintaining tear film stability,^38^^,^^39^ we observed a significant elevation of choline in SLK compared to DED. This apparent discrepancy may reflect distinct underlying mechanisms. We propose that in SLK, elevated choline is not a sign of nutritional sufficiency^40^ but a byproduct of accelerated membrane phospholipid degradation. The elevated sPLA2 activity we detected likely initiates this process by hydrolyzing PC to generate LPC. We speculate that LPC, in turn, serves as an optimal substrate for the lysophospholipase D enzyme autotaxin (ATX),^41^ which hydrolyzes LPC to release free choline and lysophosphatidic acid (LPA). Thus, the same molecule may play different roles: in DED, insufficient supply may contribute to tear film instability, as reported in previous studies,^39^^,^^42^ whereas in SLK, excessive inflammatory breakdown of membranes may release choline, marking a state of active tissue turnover. PC, a key differential metabolite in both LA and ALA metabolism, also participates in glycerophospholipid metabolism and has been shown to be related to inflammatory responses.^43^ Under the action of phospholipase A2 (PLA2),^44^ PC can produce LPC.^45^ As a biologically active lipid, LPCs have been associated with the inflammatory processes of various ocular diseases in ophthalmology.^46^^,^^47^ In our study, LPC levels were elevated in the SLK group compared to the DED group, suggesting increased PLA2 activity in the SLK group, which may promote SLK inflammation. Collectively, our study suggests that glycerophospholipid metabolism plays a crucial role in lipid signaling and mediating inflammatory responses in the pathogenesis of SLK. Understanding glycerophospholipid metabolism is vital as it may provide insights into the molecular mechanisms distinguishing SLK from DED and offer new avenues for its treatment.

Besides the aforementioned three important metabolomic pathways related to lipid metabolism, we also found several metabolites (PE, creatine, and L-acetylcarnitine) associated with inflammation responses, oxidative stress, and damage recovery that were significantly elevated. This indicates that SLK may be in a more active inflammatory state compared to DED. L-acetylcarnitine, a derivative of carnitine involved in fatty acid metabolism, is linked with DED, and under hyperosmotic stress conditions, low L-acetylcarnitine levels in DED may reduce the protective effects on the ocular surface.^17^ Our results revealed that higher levels of L-acetylcarnitine in patients with SLK compared with patients with DED. This suggests that SLK is more influenced by altered lipid metabolism and the inflammatory responses mediated by lipid mediators, rather than by damage caused by tear hyperosmolarity.

Building on our previous research, we have observed that the levels of sPLA2 in the blood of patients with SLK are significantly higher than those in healthy controls.^29^ We hypothesize that sPLA2 plays a crucial role in the lipid metabolism cascade of inflammatory responses in SLK. Thus, using Western blot, we found increased sPLA2 expression in patients with SLK compared with patients with DED and healthy controls. It has been reported that, through its catalytic and non-catalytic activities, sPLA2 plays a significant role in the pathogenesis of many inflammation-related diseases.^19^^,^^34^ On the ocular surface, sPLA2 has two main functions: significant antibacterial effects^48^ and involvement in the inflammatory process of the damaged ocular surface.^22^ The sPLA2s participate in inflammation through their enzymatic activity by releasing free fatty acids, including AA, thus initiating the biosynthesis of inflammatory mediators such as prostaglandins, thromboxanes, and leukotrienes.^49^ The levels of sPLA2 in SLK tears were significantly higher than in healthy controls, further indicating that sPLA2-mediated lipid metabolism cascade inflammatory responses play a role in the pathogenesis of SLK. This provides a research foundation for further exploration of SLK pathogenesis. Additionally, we observed that sPLA2 levels in DED were higher than in healthy controls, consistent with the findings of Wei et al.^50^ who reported elevated sPLA2 levels in patients with DED. Although our results did not show significant statistical differences, a larger sample size may be needed for further confirmation.

There are several limitations to this study. First, the sample size of this study, particularly for the SLK group (*n* = 20), remains relatively small. This is primarily attributable to the low incidence of SLK and the minute volume of tear fluid that can be practically collected from each individual, which limited our ability to perform a stratified analysis based on disease severity or duration. Furthermore, the modest cohort size constrained our ability to more comprehensively compare tear metabolomic profiles across individuals with SLK, DED, and healthy controls. Given that we have previously compared the tear metabolomics between SLK and healthy controls, this study focuses on the differences between SLK and DED. This stepwise comparison may result in incomplete and potentially partial findings because of changes in the subjects. Additionally, the selected patients with DED were primarily mild to moderate cases without obvious ocular or systemic diseases, possibly including aqueous-deficient or lipid abnormality types.^51^ Patients with early-stage MGD cannot be ruled out, so the lipid metabolism abnormalities in DED may include pre-existing lipid metabolism issues. Future multi-center studies with larger, clinically stratified cohorts are needed to validate our findings and explore the influence of disease stage and duration on tear metabolic profiles.

In conclusion, applying LC-MS/MS metabolomics analysis, we have demonstrated unique tear metabolomics in SLK that distinguish it from DED. For the first time, we reveal the glycerophospholipid metabolism abnormalities in patients with SLK, distinguishing them from patients with DED, along with the abnormal elevation of sPLA2 in SLK. These findings provide deeper support and verification for the sPLA2-PUFA-associated inflammation mechanism in SLK. This suggests that lipid metabolism abnormalities may be important pathogenic mechanisms in SLK. The results expand our understanding of the pathological mechanisms of SLK. Furthermore, this study has identified five metabolites and the combination models of them can be potential tear biomarkers for distinguishing patients with SLK from patients with DED, which provide new discriminatory evidence for the clinical diagnosis of patients with SLK and patients with DED.
